# Experience-based SEEG planning: from retrospective data to automated electrode trajectories suggestions

**DOI:** 10.1049/htl.2018.5075

**Published:** 2018-09-14

**Authors:** Davide Scorza, Gaetano Amoroso, Camilo Cortés, Arkaitz Artetxe, Álvaro Bertelsen, Michele Rizzi, Laura Castana, Elena De Momi, Francesco Cardinale, Luis Kabongo

**Affiliations:** 1 e-Health and Biomedical Applications Department, Vicomtech, Donostia-San Sebastián, Spain; 2 Dipartimento di Elettronica, Informazione e Bioingegneria (DEIB), Politecnico di Milano, Milan, Italy; 3 Claudio Munari Centre for Epilepsy and Parkinson Surgery, Niguarda Ca’ Granda Hospital, Milan, Italy

**Keywords:** medical signal processing, biomedical electrodes, diseases, data mining, neurophysiology, electroencephalography, brain, surgery, medical image processing, radiation therapy, detecting analysing similar plans, planning strategy, main tailored sets, different patient cases, experience-based SEEG planning, retrospective data, automated electrode trajectories suggestions, minimally invasive technique, multiple intracranial electrodes, epileptogenic focus, cumbersome time consuming task, current approaches, planning focus, electrode trajectory optimisation, geometrical constraints, initial electrode set, planning procedure, analyses retrospective planning data, average trajectories, anonymised patients, 30 exploratory patterns, 61 mean trajectories, average brain space, test set, manual trajectories, insertion angle

## Abstract

StereoElectroEncephaloGraphy (SEEG) is a minimally invasive technique that consists of the insertion of multiple intracranial electrodes to precisely identify the epileptogenic focus. The planning of electrode trajectories is a cumbersome and time-consuming task. Current approaches to support the planning focus on electrode trajectory optimisation based on geometrical constraints but are not helpful to produce an initial electrode set to begin with the planning procedure. In this work, the authors propose a methodology that analyses retrospective planning data and builds a set of average trajectories, representing the practice of a clinical centre, which can be mapped to a new patient to initialise planning procedure. They collected and analysed the data from 75 anonymised patients, obtaining 30 exploratory patterns and 61 mean trajectories in an average brain space. A preliminary validation on a test set showed that they were able to correctly map 90% of those trajectories and, after optimisation, they have comparable or better values than manual trajectories in terms of distance from vessels and insertion angle. Finally, by detecting and analysing similar plans, they were able to identify eight planning strategies, which represent the main tailored sets of trajectories that neurosurgeons used to deal with the different patient cases.

## 1 Introduction

Drug-resistant focal epilepsy represents a potentially treatable disorder, once the anatomical originating area is defined (so-called epileptogenic zone, EZ). When the neuroimaging is poorly supporting the definition of the EZ, an invasive monitoring can be considered. StereoElectroEncefaloGraphy (SEEG) is a percutaneous surgery that allows recording the electrical activity of the brain, through surgically implanted intracranial electrodes [1]. The surgical step of SEEG includes the planning of the electrodes to be placed [2]. Similar to other minimally invasive neurosurgeries, trajectories must avoid vessels, provide a small probe-skull angle at the entry point, and reach the correct targets. Recent research in neurosurgery has been focusing on the optimisation of trajectories in order to decrease the planning time and improve the procedure safety and accuracy. In [3], the authors implemented a method for automatic trajectory proposal that computes the risk based on a two-step approach which combines a multi-objective optimisation and fuzzy logic. Specifically, in Deep Brain Stimulation (DBS), in [4] the authors improved previous methodologies by optimising both the trajectory and the stimulation point, by the use of an anatomo-clinical atlas and an estimation of the volume of tissue activated. However, with respect to DBS or general key-hole neurosurgery, which requires accurate targeting of reduced brain zones, SEEG requires a higher number of trajectories aimed to record different cerebral regions. Automated planning in this field has been focusing on the optimisation of electrode trajectories based on requirements such as the maximisation of the distance from vessels, the minimisation of entry angle, increase of GM sampling and conflict avoidance. Optimisation approaches usually involve one electrode at a time, except for conflict resolution strategies where the whole set of trajectories is considered. In [5] the authors proposed a method able to optimise the trajectories and maximise the grey matter volume recorded. However, their study was limited to three electrodes at a time. Only Sparks *et al.* [6] propose a method which computes the minimum number of electrodes able to cross all the required regions selected by the surgeon. Nevertheless, this method has the drawback of being very dependent on the atlas used and could lead to solutions that may not be aligned with real clinical decisions. In addition, as shown in a recent study [7], the planning strategy of different centres may differ due to the hardware used for electrode placement, the imaging protocol and the centre experience. Therefore, even if the safety and efficacy requirements are similar, there are no standardised rules that guide all clinical centres and a more tailored approach may provide a better answer to specific centre requirements. In Scorza *et al.* [8], electrodes are manually initialised by surgeons by placing rough entry and target points (respectively, EP and TP), while the atlas is only used to maintain the initial anatomical zones during optimisation. In this way, the initialisation guarantees to respect the clinical practice but requires a manual intervention that may be time consuming.

In this work, we propose a new methodology able to analyse retrospective data from successful SEEG implants and extract the most common trajectories used for the exploration of specific brain zones in a given medical centre. The main assumption of this work is that despite SEEG is a patient specific surgery based on individual anatomy and brain activity, it is possible to identify exploration strategies and trajectories that are commonly used to explore multiple brain areas and to provide an adapted model for the centre practice. In this way, our system can suggest an initial plan for a new patient, which the neurosurgeon can further adjust manually or be adapted to the specific anatomy thanks to an optimisation framework as the ones previously described. As far as we know, this is the first attempt to model SEEG practice combining surgeon knowledge with specific centre retrospective data. The final application will provide trajectory suggestions aligned with the clinical experience, and may be a valid assistant, especially for junior surgeons. Preliminary experiments show that, after optimisation, the initialised trajectories reach similar values in terms of safety that those that have been manually planned (MP) by neurosurgeons. Finally, we were able to cluster the trajectories into various planning strategies that have been positively recognised by a surgeon as commonly adopted in the centre.

## 2 Methodology

SEEG is a very tailored surgery, influenced by the specific anatomy and the particular case of each patient. Most of the time, the procedure does not require to reach a precise target. Instead, a set of regions needs to be sufficiently sampled to identify the epileptogenic network. Nevertheless, it is possible to identify exploratory patterns or sets of trajectories aimed to explore the same brain regions in different patients. With the aim of identifying and modelling similar trajectories, the clinical target (target zone between the ones traversed by a single trajectory) needs to be extracted. Actually, the electrode end-point does not necessarily match the clinical target, it can be any of the zones that it crosses, and clinical knowledge is required here to clearly define the target. Hence, we can associate a generic trajectory descriptor $d = \lsqb A\semicolon \; B\rsqb $, where *A* and *B* represent the entry and the clinically relevant target regions, respectively. In this work, retrospective data are collected and analysed with the aim of modelling the clinical practice of a centre and use this knowledge for the initialisation of new plans. A plan $p = \lcub {\rm t}{\rm r}_1\comma \; \ldots \comma \; {\rm t}{\rm r}_E\rcub $ is a set of trajectories aimed to explore a set of brain zones, with *E* being a variable number of electrodes depending on the case.

### 2.1 Problem statement

Given the MRI image of a new patient and a planning strategy selected by the surgeon, the goal of the system is to provide a set of trajectories based on surgeons’ past experience adapted to current patient specific anatomy. The planning strategy defines a reduced set of trajectories aimed for the exploration of specific brain regions. Those trajectories will be then modified or optimised to adapt them to the specific patient anatomy.

### 2.2 Solution strategy

The following steps were conducted for the implementation of the proposed system:

- *Trajectory descriptor and exploratory patterns:* analysis of retrospective cases in subject space, identification of similar trajectories among patients and their normalisation on an average brain space.
- *Mean trajectories definition (mT):* spatial clustering in the average brain space of similar trajectories to produce mean trajectories mT for the exploration of specific brain regions.
- *Planning strategies definition (cl):* analysis of the spatial relationship between mean trajectories and their clustering, based on the macro-anatomical regions that they explore.
- *Plan adaptation:* mapping of the selected planning strategy from a common average brain space to the subject space.

Finally, the trajectories obtained may be optimised similarly as described in [8].

### 2.3 Trajectory descriptor and exploratory patterns

We collected the retrospective data of *N* patients who successfully underwent SEEG procedure. The inputs for our analysis are an MRI-t1 image and the original trajectories planned by the surgeon in subject space. All data have been processed by the Freesurfer (FS) pipeline [9], which co-registers the patient with the MNI-305 space and labels the different brain zones using a probabilistic atlas segmentation [10]. For each patient, a plan $p_j$ with $j = 1\comma \; \ldots \comma \; N$ is assigned, where trajectories ${\rm t}{\rm r}_{i\comma j}$ have been MP by surgeons and are defined by the entry and target point coordinates. Trajectories were regularly sampled with a method similar to the one described in [11]. For each ${\rm t}{\rm r}_{i\comma j}$, we defined a descriptor $d_{i\comma j} = \lsqb Z_{{\rm EP}}^a \semicolon \; Z_{{\rm TP}}^b \rsqb $, where $Z_{{\rm EP}}$ and $Z_{{\rm TP}}$ are groups containing the labels of the brain zones that are considered as meaningful entry and target zones, respectively ($Z_{{\rm EP}}^a \in Z_{{\rm EP}}$ and $Z_{{\rm TP}}^b \in Z_{{\rm TP}}$). This definition of $Z_{{\rm EP}}$ and $Z_{{\rm TP}}$ resulted from the analysis of the most explored zones in our samples, their anatomical positions, and surgeons suggestions. Finally, sets of similar trajectories ${\rm p}{\rm t}_{a\comma b} = \lcub {\rm t}{\rm r}_{i\comma j}\rcub \comma \; \; \forall {\rm t}{\rm r}_{i.j}\; {\rm if}\; d_{i\comma j} = \lsqb Z_{{\rm EP}}^a \semicolon \; Z_{{\rm TP}}^b \rsqb $ were built from all patient data and represent exploratory patterns to explore the regions $\lsqb Z_{{\rm EP}}^a \semicolon \; Z_{{\rm TP}}^b \rsqb $.

### 2.4 Mean trajectory definition

**Fig. 1:**
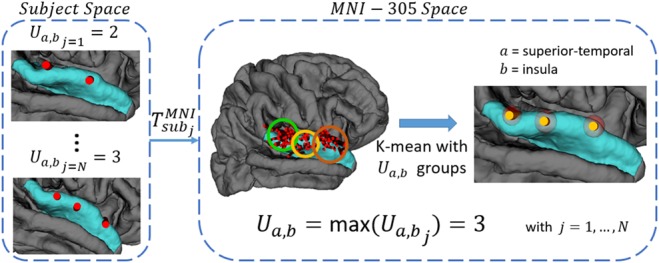
Mean trajectories for a single pattern $pt_{a\comma b}$ obtained with k-means algorithm: insular exploration (region b) is usually performed from the superior-temporal (region a), with a maximum of three electrodes in the same plan $U_{a\comma b} = 3$

Once similar trajectories have been grouped in subject space, their coordinates were transformed into the MNI-305 space by using the registration matrix provided by the FS pipeline. Since we based our analysis on an anatomical atlas, we must assume that, due to their size, some regions are explored with several electrodes for higher coverage. Therefore, for a given plan $p_j$ we may find a variable number of trajectories ${\rm t}{\rm r}_{i\comma j}$ with the same descriptor ${\rm p}{\rm t}_{a\comma b}$. To keep the spatial relationship between those trajectories, we used a k-means algorithm to automatically cluster entry and target coordinates into $U_{a\comma b}$ groups and generate a set of mean trajectories ${\rm mT}_{a\comma b}^u $, where $u = 1\comma \; \ldots \comma \; U_{a\comma b}$. The number of groups $U_{a\comma b}$ is defined as the maximum number of trajectories aimed to explore the same regions *a* and *b* in a single plan among all plans $p_j$, $j = 1\comma \; \ldots \comma \; N$. To avoid an erroneous definition of groups due to particular cases, an ${\rm mT}_{a\comma b}^u $ is considered significant only if contains at least 5% of trajectories described by ${\rm p}{\rm t}_{a\comma b}$. Otherwise, the maximum number of clusters $U_{a\comma b}$ is decreased by 1 and the k-means algorithm applied iteratively. Fig. 1 shows an example of the procedure for trajectories commonly used to explore the insular region. Finally, we obtained a set of mean trajectories ${\rm mT} = \lcub {\rm mT}_{a\comma b}^u \rcub $ where $a \in Z_{{\rm EP}}$, $b \in Z_{{\rm TP}}$ and $u = 1\comma \; \ldots \comma \; U_{a\comma b}$, composed of couples of mean entry and target coordinates and their standard deviations.

### 2.5 Planning strategies

**Fig. 2:**
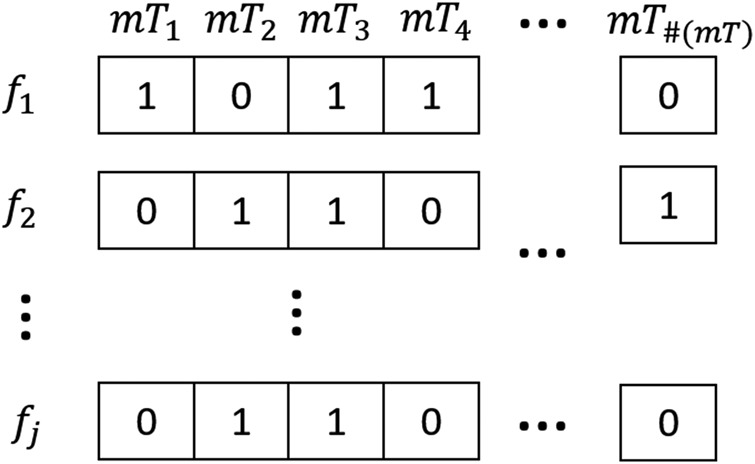
Binary vector representation $f_j$ for each plan $p_j$, representing the presence or the absence of a mean trajectory. The Jaccard distance is computed to measure the dissimilarity between binary vectors

To find a high-level feature describing a precise planning strategy, the different plans were compared and clustered. Since we defined a set of mean trajectories mT from MP trajectories ${\rm t}{\rm r}_{i\comma j}$, we can define and assign to each plan $p_j$ a new binary descriptor $f_j$ of fixed length $\# \lpar {\rm mT}\rpar $, which contains only Boolean values and represent the presence or the absence of a mean trajectory ${\rm m}{\rm T}_y$, with $y = 1\comma \; \ldots \comma \; \# \lpar {\rm mT}\rpar $ in a plan $p_j$. Since the plans can be hierarchically connected (e.g. one can be the composition of others), we opted for a hierarchical clustering method which operates on the basis of an empirical coefficient of similarity (the Jaccard distance) computed over the descriptors $f_j$ (Fig. 2).

By the analysis of the generated dendogram, we grouped the plans into *G* clusters by the selection of a cut-threshold. Finally, for each group $g \in G$, we defined a new binary descriptor ${\rm c}{\rm l}_g$ of length $\# \lpar {\rm mT}\rpar $ and we set ${\rm cl}_g^y = {\rm True}$ if at least $\# \lpar f_j^y = {\rm True}\rpar \gt = 2$, $\forall f_j$ in *g*. The new descriptor ${\rm c}{\rm l}_g$ codifies a set of mean trajectories to explore macro-regions of the brain and represents a planning strategy.

### 2.6 Clinical scenario and validation

To construct our model we used 75 anonymised patient data provided by Niguarda Hospital (Milan, Italy), for a total of 1100 trajectories. For each patient, MR images were acquired using the hospital system $1.5T$ (Intera Achieva, Philips Medical System, The Netherlands, T1 3D FFE sagittal images, $0.90\, {\rm mm} \times 1.07\, {\rm mm} \times 0.90\, {\rm mm}$ voxel dimensions, without any inter-slice gap, then reconstructed and reformatted on the axial plane with the $560 \times 560 \times 220$ matrix, $0.45\, {\rm mm} \times 0.45\, {\rm mm} \times 0.9\, {\rm mm}$ voxel dimensions). The FS pipeline was used to obtain the cortical reconstructed surface and the probabilistic atlas segmentations (the Desikan–Killiany atlas, with 75 labels per hemisphere, was used in this study). The pipeline also provides the affine registration matrix used to map patients and trajectories from the subject space to the average space (MNI-305). As a preliminary validation of our method, we used a test set composed of ten patients that were not included to build our model. For each patient's plan, we identified the MP trajectories corresponding to mean trajectories ${\rm m}{\rm T}_y$ and mapped them to the subject's space generating and initialised plan (IP). Hence, we computed the Euclidean distances $d_{{\rm ep}}^{\alpha \comma \beta } $ and $d_{{\rm tp}}^{\alpha \comma \beta } $ between entry and target point coordinates, respectively, where $\alpha \comma \; \beta $ are two corresponding trajectories between the mapped mean trajectories and those planned by the surgeon. We considered that a trajectory has been correctly mapped when $d_{{\rm ep}}^{\alpha \comma \beta } \le 2\sigma \lpar {\rm mT}_y^{{\rm ep}} \rpar $ and $d_{{\rm tp}}^{\alpha \comma \beta } \le 2\sigma \lpar {\rm mT}_y^{{\rm tp}} \rpar $. The value of $\sigma $ varies based on the trajectories used to define the average ${\rm m}{\rm T}_y$. This metric provides a measure of the generalisability of trajectories mT. However, the mapped mean trajectories may not comply with clinical criteria (e.g. safe distance to vessels), and therefore they need to be optimised to produce valid initial plans and adapt to the specific patient anatomy. For the optimisation, we used the method presented in [8] and verified the compliance in terms of distance from vessels and insertion angle. We evaluated initial quantitative values comparing manual planned (MP) trajectories, the corresponding initialised trajectories (IP), and their optimised solution OMP and OIP, respectively. Finally, the groups obtained by the hierarchical clustering have been presented and qualitatively evaluated by a surgeon. Results are reported in the following section.

## 3 Results

### 3.1 Exploratory patterns and mean trajectories

The analysis of the planned trajectories based on the Desikan–Killiany atlas reduced the possible target regions used to classify the trajectories. We did not take into account the white matter, while other structures such as ventricles, brain stem or cerebellum, where automatically excluded since they were classified as outliers. Following the clinician's advice, electrodes crossing the *insula* and ending in the *putamen* were included in the Insular pattern. In the same way, we consider as a single target point the *hippocampus* and the *para-hippocampus*, since it is not possible to explore the last without crossing the *hippocampus*, and on the other side by prolonging the trajectory we easily reach the *para-hippocampus*, increasing the recorded information. Finally, since our data had a poor representation of occipital trajectories, we joined the occipital zones of *lingual cortex*, *cuneus*, *pre-cuneus* and *pericalcarine*, assuming that the spatial distribution of those was more important than the specific description pattern in terms of entry and target zones.

**Fig. 3:**
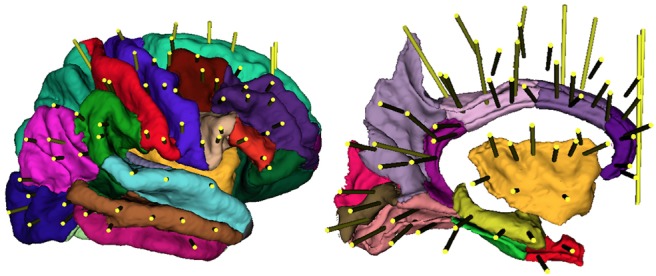
Sagittal view of the mean trajectories computed in the average space: dimensions have been enlarged for visualisation purposes

By grouping the trajectories with the reduced descriptors presented in Section 2.3, we found 30 exploratory patterns ${\rm p}{\rm t}_{a\comma b}$, for a total of 61 mean trajectories mT that represent the mean coordinates of the most representative trajectories in our data. Fig. 3 shows the mean trajectories in the average space.

**Fig. 4:**
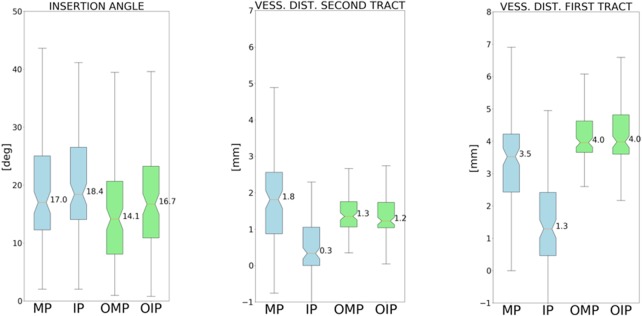
Comparison of quantitative indices between manual planning (MP), initialised planning (IP) trajectories and their optimised versions (OMP and OIP). The mean values of insertion angle and distance from vessels (first and second tracts as reported in [8]) present no statistical difference between OIP and OMP

To test the viability of our method, we mapped the recognised trajectories on 10 patients that were not included in the initial database as explained in Section 2.6, for a total of 95 initialised trajectories. By computing the initial Euclidean distance with their corresponding MP trajectories at EP and TP in subject space, we found that the 90% of the initialised ones satisfy the criteria presented in Section 2.6. Finally, to test the viability of our initialisation method, we compared the values of the distance from vessels at the entry points, distance from vessels in the second tract and insertion angle before and after the optimisation for both groups (MP and IP). The results are shown in Fig. 4.

Even if the mapped trajectories (IP) could not be considered safe in terms of indices, the optimisation performed provides a better solution (OIP group) with respect to the MP trajectories and a comparable solution with respect to the optimised MP (OMP) ones, making this a valid method to initialise an optimisation strategy provided by an automated planner. No statistical difference has been found between the indices of the two groups OMP and OIP after optimisation.

### 3.2 Planning clustering and strategies definition

**Fig. 5:**
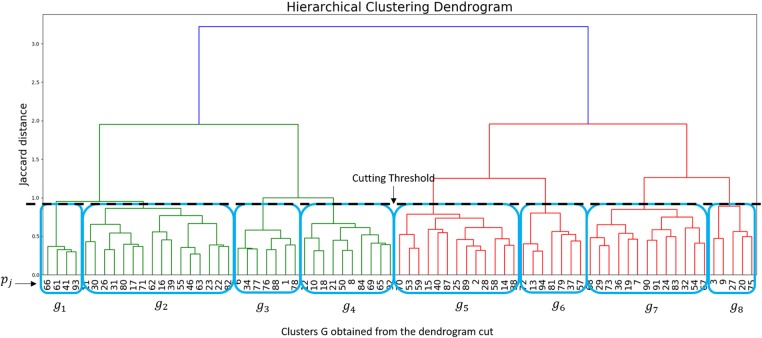
Dendrogram obtained by the hierarchical clustering performed using the Jaccard distance. The cutting threshold defined eight groups

The hierarchical clustering method applied and the cutting-threshold chosen led to eight different clusters composed by similar plans. The cut-threshold has been chosen empirically, in order to balance the number of groups and its components (Fig. 5).

**Fig. 6:**
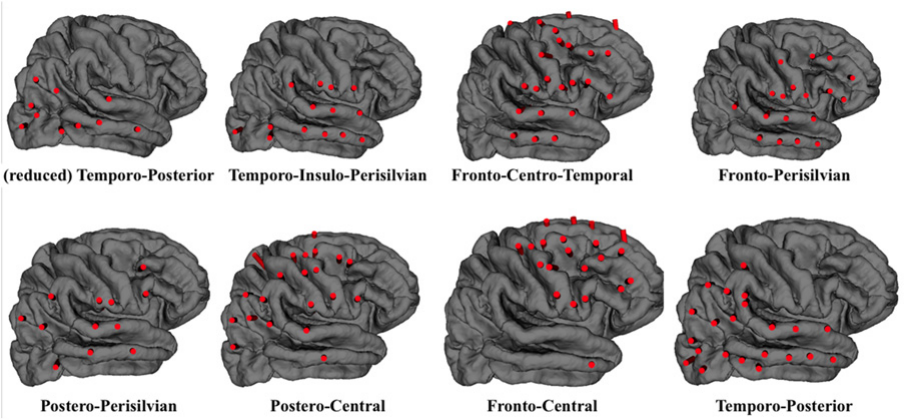
Lateral view of the eight clusters obtained, with the labels defined according to the surgeon suggestions

Therefore, for each cluster obtained we selected those trajectories which appear in at least two plans and generate the planning strategies ${\rm c}{\rm l}_g$. The clusters were evaluated by a neurosurgeon, who recognised the main trends in the trajectories proposed. Therefore, we were able to name each cluster, as reported in Fig. 6.

Since SEEG is a patient-specific procedure, the clusters obtained do not completely match with actual patient plans. In addition, some of the clusters (e.g. *Fronto-central*) result to be over-populated, since we preferred a reduced number of clusters with more trajectories rather than more groups to be combined. From a usability point-of-view, we considered that removing trajectories from a suggested strategy would be easier for the surgeon in order to adapt the plan for a specific patient than to combine different cluster and then refine the plan. However, they seem to be a good representation of the planning strategies adopted in the centre to explore specific macro-areas of the brain. Notice that the results have been obtained from a reduced set of data, with an unbalanced distribution of plans. However, the system was able to group the plans into clinically meaningful clusters, that can be used as an initial starting point for a new patient planning. By the use of those clusters, the clinician should only remove or add few specific trajectories, while the rest would be directly mapped to the patient anatomy. Nonetheless, the use of a cluster is not mandatory, since there may be specific patients where the direct choice of single trajectories from the ${\rm mT}$ set would provide a faster initialisation.

## 4 Conclusion

In this work, we presented a new methodology that merges clinical knowledge with the analysis of retrospective data of a given clinical centre. The approach presented has been able to identify and model the most used trajectories, and define specific planning strategies by clustering similar plans. To the best of our knowledge, this is the first application that provides a trajectory initialisation method adapted to a single centre strategy based on its retrospective data analysis. The final application allows to easily visualise and selects the most common trajectories, and cluster electrodes commonly used for a specific exploration. Preliminary results show that mean trajectories can be successfully mapped to patient plans that were not included to build our model, and provide a meaningful initialisation for an automated planner as the work presented in [8]. The clusters obtained have been positively recognised by surgeons as exploratory strategies used in their centre and we were able to map 90% of the trajectories. Finally, those initialised trajectories have been correctly optimised by the automated planner, adapting to the subject anatomy and reaching quantitative results comparable or superior to MP trajectories, in terms of safety. An extended validation with surgeons is still needed to assess the viability of those trajectories. Current limitations of this approach are represented by the need of FS pipeline and of a large amount of data. Since the trajectories extracted represent the most common trajectories used in the centre, a new patient plan would be unlikely initialised completely by a chosen set and surgeons will still have to manually plan few trajectories depending on specific patient requirements (e.g. lesions). Nonetheless, the method presented provides a model of the clinical practice mostly based on the data provided by the centre. The database constructed for this work will store new patient information and their planned trajectories, allowing our model to continuously adapt while new patients are added. To improve our model, especially regarding the planning strategies proposed, Diffusion Weighted Imaging and tractography may be used to identify specific brain networks and generate more specific electrode clusters to map them. Similarly, in the future, we would like to include other functional imaging techniques as functional-MRI and/or EEG-signals. Future work will be focused on the generalisation of this methodology and to provide a more complete validation.
